# Ab interno implantation of glaucoma drainage devices tubes in the posterior chamber

**DOI:** 10.1186/s12886-020-1329-1

**Published:** 2020-02-22

**Authors:** Javier Moreno-Montañés, Concepción Guirao-Navarro, Francisco Argüeso

**Affiliations:** 1 Department of Ophthalmology, Clínica Universidad de Navarra, Universidad de Navarra, Avda. Pio XII, 36-31008 Pamplona, Navarra Spain; 2 Clínica Virgen de Luján, Sevilla, Spain

**Keywords:** Glaucoma drainage device, Posterior chamber, Pseudophakic eye, Corneal edema

## Abstract

**Background:**

Glaucoma drainage device (GDD) implantation in the anterior chamber are associated with corneal complications. We describe a novel technique to implant GDD tubes in the posterior chamber of pseudophakic eyes.

**Methods:**

Ten patients with glaucoma who required GDD tube implantation were included.

**Results:**

The new technique begins with the passage of one of two straight needles existing at each end of a 10–0 Polypropylene suture through the GDD tube. A 23-gauge needle then is inserted at an angle 180° away and passed from the anterior to the posterior chamber and finally through the sclera. The two suture straight needles from the 10–0 Polypropylene suture are positioned in the lumen of the 23-gauge needle. The 23-gauge needle is then extracted from the eye by passing the 2 needles through the lumen. The suture remains inside the posterior chamber, and the tube is inserted into the posterior chamber by pulling on the suture from the other side. No intra-operative complications were found such as bleeding, vitreous tube placement, bent tubes, etc.

**Conclusions:**

This surgical procedure to implant a tube into the posterior chamber of the pseudophakic eyes is uncomplicated and facilitates the insertion of the flexible tube into the posterior chamber. This eliminates the tendency of the tube to enter the vitreous as the tube is always placed in the posterior chamber away from the cornea.

**Trial registration:**

Current Controlled Trials ISRCTN14276553 (31th May, 2019) Retrospectively registered.

## Background

Glaucoma drainage device (GDD) implantation is one of the most commonly performed surgical procedures for treating refractory glaucoma. However, GDD implantation is associated with complications, one of the most undesirable of which is long-term corneal decompensation. Corneal edema can result from multiple factors, but one is mechanical endothelial damage from the tubes in the anterior chamber. These tubes potentially can cause damage when they are near the cornea or in contact with the cornea during blinking, ocular movements, or eye rubbing, among others [1]. A proposed technique to move the tube away from the cornea requires tubal implantation into the vitreous cavity through the sulcus. However, this requires pars plana vitrectomy, which also has risks, especially if the vitreous must be removed completely [2]. Another possibility is tubal implantation into the posterior chamber between the iris and the intraocular lens in pseudophakic eyes. However, this technique is not always easy if the tube is inserted from outside the eye, because it may collide with the intraocular lens (IOL) or iris, be bent under the iris, or go into the vitreous.

We report an uncomplicated and straightforward procedure to implant a silicone tube in the posterior chamber in pseudophakic patients as an alternative to avoid tubal complications in the anterior chamber or vitreous.

## Methods

**Fig. 1:**
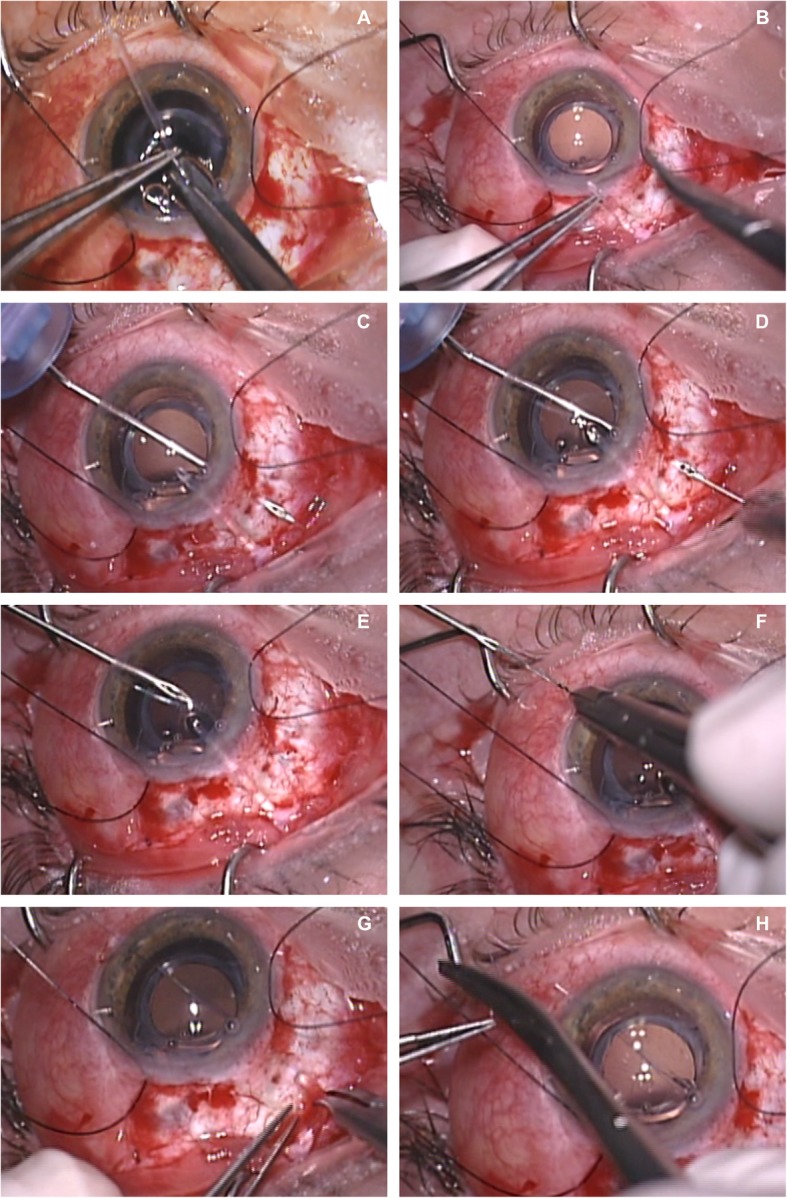
Surgical technique of implantation of a glaucoma drainage device (GDD) tube into the posterior chamber. After cutting the tube (**a**), a needle with a Prolene suture (**b**) is inserted through the tube; a 23-gauge needle then is inserted through a clear corneal incision into the anterior chamber and advanced to the posterior chamber and through the sclera and eyeball (**c**). The two straight needles with the Prolene sutures are introduced into the lumen of the 23-gauge needle (**d**), and the 23-gauge needle is removed (**e**). The two needles attached to the Prolene sutures are removed from the 23-gauge (**f**) needle lumen; the GDD tube then is inserted simultaneously as the Prolene suture is pulled out (**g**). Finally, one of the ends of the Prolene sutures is cut out of the eye, and then the other end is pulled to completely remove the suture from the inside of the eye, since the suture is not knotted to the tube but only goes through the tube (**h**)

A conjunctival peritomy is performed in a quadrant and the GDD plate is sutured 8 to 10 mm from the limbus as in the standard GDD implantation procedure. Initially, we recommend injecting hyaluronate into the posterior chamber to enlarge the chamber to facilitate movement of a 23-gauge needle in the posterior chamber. The new surgical technique of GDD tube implantation begins first with shortening the tube to an appropriate length to facilitate visualization through the pupil. (Fig. 1a). The length of the tube is calculated manually and is cut with scissors so that the bevel is - if possible- positioned somewhat laterally. One of the two needles at each end of a 9 or 10 Prolypropylene suture perforates the final part of the tube and is extracted, maintaining the suture through the tube. (Fig. 1b). A clear corneal incision is made on the opposite side from the site of the tubal implantation, and a 23-gauge needle is introduced through this incision into the anterior chamber, advanced into the posterior chamber, and passed through the sclera until the tip of the needle exits the eyeball (Fig. 1c). The two straight needles attached to the Prolene sutures are inserted into the lumen of the 23-gauge needle (Fig. 1e) and the 23-gauge needle (Fig. 1e) and the two needles are pulled out with the suture through the eyeball (Fig. 1f). The scleral incision that was created previously can be used to insert the tube and the sutures that pass through this incision. The tube then is simply inserted through the incision and the two Prolene sutures are pulled together to allow the tube to enter easily and be inserted into the posterior chamber (Fig. 1g). This traction of the Prolene sutures prevents the tube (when pushed only from the outside) from entering the vitreous cavity or from bending when there is friction between the iris or ciliary body. Once the tube is in place, one of the Prolene sutures is cut and removed from the eye by pulling it from either side (Fig. 1h). All patients provided informed consent before this surgery, which adhered to the Declaration of Helsinki.

## Results

We performed this procedure in 10 cases (Table 1) without complications. The GDD tubes were well positioned. In all the cases, a Baerveldt GDD was implanted. The end of the tube was seen at the periphery of the pupil without nearing the center of the cornea and without any optical effect. No particular intra-operative difficulties or complications such as bleeding, vitreous tube placement, bent tubes, etc. were observed. No iris damage or pigment dispersion was found. Finally, after the follow-up period (Table 1), no tube displacement or extrusion, or iris alteration were found. The endothelium data are not analysed for several reasons: the surgical technique is different (in some cases only the tube is implanted and in others phacoemulsification is also performed); some eyes were previously operated for glaucoma and others not; and the follow-up time is different in some cases than in others. However, no corneal edema was found after the follow-up period.

**Table 1:** Characteristics and follow-up of the 10 patients with glaucoma with changes in the intraocular pressure values using our surgical approach

Case	Age (years)	Diagnosis	Follow-up (months)	Previous glaucoma surgeries	Other surgical procedures	IOP before surgery	IOP after follow-up	Medicine changes	Other ocular characteristics
1	86	OAG	48	Two trab,	Phaco	24	12	-3	–
2	69	PSX	36	One trab, Ahmed	Phaco	26	15	−4	Advanced glaucoma
3	69	Uveític glaucoma	46	One trab	Phaco	32	10	-4	Advanced glaucoma
4	77	PSX	6	One trab	Phaco	27	11	−3	Advanced glaucoma
5	69	OAG	12	One trab	Phaco	20	10	−2	High myopia
6	90	PSX	24	Two trabs	Phaco, IOL dislocation	18	10	−3	Advanced glaucoma and ARMD
7	56	Traumatic glaucoma	32	Ex-Press	Phaco	28	20	−1	Advanced glaucoma, angle recession
8	32	ICE (ACG)	36	One trab	Phaco	42	17	−4	Ptosis
9	74	PSX	18	One trab	Phaco, IOL dislocation	28	9	−2	Terminal glaucoma
10	58	Neovascular glaucoma	48	None	Phaco	50	14	−5	Terminal glaucoma, anti-VEGF injections, and retinal panphotocoagulation

*OAG* open-angle glaucoma, *Trab* trabeculectomy, *Phaco* cataract surgery with phacoemulsification, *ARMD* exudative age-related macular degeneration, *PSX* pseudoexfoliative glaucoma, *ICE* iridocorneal endothelial syndrome, *IOL* intraocular lens, *ACG* angle-closure glaucoma; VEGF, vascular endothelial growth factor

## Discussion

Tube shunts in the anterior chamber are associated with progressive endothelial cell loss [3]. Persistent corneal edema attributable to the GDD tube was reported to be almost 12% in the Ahmed Baerveldt Comparison Study after 5 years of follow-up [4]. Using anterior-segment optical coherence tomography, Tan et al. found that the tubal-corneal distance significantly affects the corneal endothelial cell density, i.e., the shorter the distance, the more severe the endothelial cell loss [5]. Therefore, maximization of the distance between the tube and the cornea is important, especially in eyes with other risk factors associated with endothelial damage but without performing a posterior vitrectomy. The current technique is easy to perform; the tube is inserted adequately behind the iris but remains visible through the pupil and is a safe distance from the cornea. We have performed other surgical approaches to insert the tube into the posterior chamber, but in our hands, the current technique is the simplest of all the approaches to achieve that end [6].

Our surgical procedure has other potential advantages in that it can be performed with different GDDs with a 23-gauge tube (Ahmed or Baerveldt), it can be performed simultaneously with cataract surgery or penetrating keratoplasty or Descemet membrane endothelial keratoplasty (DMEK), or to change the tube from the anterior to the posterior chamber in cases with rapid endothelial cell loss. The tube also is well positioned and avoids continuous rubbing against the iris, which can lead to reduced iris depigmentation or intraocular inflammation. The characteristics of this technique that distinguish it from other approaches are: first, the location of the tube is determined ab interno with the 23-gauge needle exiting the posterior chamber to the outside of the eyeball. The horizontal white-to-white distance and posterior chamber depth in pseudophakic eyes differ among patients, and there are anatomic variations [7]. This ab interno approach is similar to that previously described by Camejo et al. [8]. However our technique differs in the method of introducing the tube; these authors introduce it by pushing the tube [8]. In our technique the tube is pulled with the suture from inside the eye, which eliminates the tendency of the tube to enter the vitreous cavity; it also prevents the flexible tube from bending when obstructing the ciliary body, IOLor iris. A disadvantage is the additional cost of the Prolene sutures for this surgical approach.

## Conclusion

This surgical procedure is easy to perform and it facilitates insertion and movement of the flexible tube into the posterior chamber. The procedure can be performed with all GDD implantations; it is not time-consuming and is safer for the endothelium than anterior chamber tube implantation. Although we did not experience complications in these 10 cases, a study that includes more patients and surgeons is required to determine the safety of this innovative surgical approach.

## Data Availability

The data and material are available from the Department of Ophthalmology, University of Navarra (J. Moreno-Montañés).

## Abbreviations

DMEK: Descemet membrane endothelial keratoplasty
GDD: Glaucoma drainage device

## References

[1] Tello C., Espana E. M, Mora R., Dorairaj S., Liebmann J. M, Ritch R. (2007). Baerveldt glaucoma implant insertion in the posterior chamber sulcus. British Journal of Ophthalmology.

[2] Sidoti PA, Mosny AY, Ritterband DC, Seedor JA. Pars Plana Tube Insertion of Glaucoma Drainage Implants and Penetrating Keratoplasty in Patients with Coexisting Glaucoma and Corneal Disease. Vol 108.; 2001. https://ac.els-cdn.com/S0161642001005838/1-s2.0-S0161642001005838-main.pdf?_tid=c0ecb240-0a30-4f84-812e-db67764acb34&acdnat=1534674046_0b94457da4db1bf7ebc295309fdf9a39. Accessed August 19, 2018. 10.1016/s0161-6420(01)00583-8 11382628

[3] Lee E-K, Yun Y-J, Lee J-E, Yim J-H, Kim C-S (2009). Changes in corneal endothelial cells after Ahmed Glaucoma valve implantation: 2-year follow-up. AJOPHT..

[4] Budenz Donald L., Feuer William J., Barton Keith, Schiffman Joyce, Costa Vital P., Godfrey David G., Buys Yvonne M., Budenz Donald, Gedde Steven J., El Sayyad Fouad, Herndon Leon, Godfrey David, Fellman Ronald, Robinson James, Dueker David, Riedel Patrick, Samuelson Thomas, Barton Keith, Puertas Renata, Chew Paul, Aquino Cecilia, Solish Alfred M., Buys Yvonne, Trope Graham, Brandt James D., Lim Michele, Law Simon, Costa Vital, Sarkisian Steve, Chopra Vikas, Francis Brian, Meallet Mario, Varma Rohit, Netland Peter, Salim Sarwat, Feldman Robert, Bell Nicholas, Chen Philip, Heuer Dale, Singh Kuldev, Wright Martha, Budenz Donald L., Feuer William J., Schiffman Joyce C., Barton Keith, Budenz Donald L., Feuer William J., Feuer William J., Schiffman Joyce C., Shi Wei, Ajuria Luz, Silva Yolanda (2016). Postoperative Complications in the Ahmed Baerveldt Comparison Study During Five Years of Follow-up. American Journal of Ophthalmology.

[5] Tan AN, Webers CAB, Berendschot TTJM (2017). Corneal endothelial cell loss after Baerveldt glaucoma drainage device implantation in the anterior chamber. Acta Ophthalmol.

[6] Moreno-Montañés J, Fantes F, García-Gómez P (2008). Polypropylene suture-guided valve tube for posterior chamber implantation in patients with pseudophakic glaucoma. J Cataract Refract Surg.

[7] Reinstein DZ, Archer TJ, Silverman RH, Rondeau MJ, Jackson Coleman D. Correlation of Anterior Chamber Angle and Ciliary Sulcus Diameters with White-to-White Corneal Diameter in High Myopes Using Artemis VHF Digital Ultrasound. Vol 25.; 2009. https://www.ncbi.nlm.nih.gov/pmc/articles/PMC2649749/pdf/nihms78851.pdf. Accessed August 19, 2018. 10.3928/1081597x-20090201-03 PMC2649749 19241769

[8] Camejo L, Noecker R (2008). Ab Interno sulcus placement of Glaucoma tube implants. Ophthalmic Surg Lasers Imaging.

